# Vitamin D Deficiency and Immune Disorders in Combined Endocrine Pathology

**DOI:** 10.3389/fendo.2018.00600

**Published:** 2018-10-09

**Authors:** Yuliia I. Komisarenko, Maryna I. Bobryk

**Affiliations:** Endocrinology Department, Bogomolets National Medical University, Kyiv, Ukraine

**Keywords:** diabetes mellitus, autoimmune thyroiditis, 25-OH D, vitamin D_3_ supplementation, immune disorders, cytokine profile, Chernobyl disaster

## Abstract

**Introduction:** Combined endocrine pathology is a serious healthcare problem in Ukraine. This prospective study assessed the blood levels of 25-hydroxyvitamin D [25(OH)D] and markers of immune function in response to vitamin D intervention in patients with type 1 and type 2 diabetes mellitus (T1DM and T2DM, respectively) and autoimmune thyroiditis (AIT).

**Objective:** This study evaluated the relationship between the metabolic and immune status of DM + AIT patients with respect to their vitamin D status and changes after vitamin D_3_ supplementation.

**Material and Methods:** Patients with type 1 or type 2 DM in combination with AIT and decreased circulating levels of 25(OH)D were divided into two groups of 30 patients each. All patients with AIT were euthyroid and receiving hormonal replacement therapy. The levels of carbohydrate and fat metabolism markers, Immunologic markers, namely, Th1-type cytokines [interferon (IFN)-γ, tumor necrosis factor (TNF)-α, interleukin (IL)-2, IL-6, IL-12], Th2-type cytokines (IL-4, IL-5), IL-10, and IL-17 were measured before and after vitamin D_3_ supplementation. The vitamin D status was assessed according to the level of 25(OH)D.

**Results:** Patients with combined endocrine disorders (DM and AIT) with a decreased vitamin D status had significantly increased background concentrations of Th1-type cytokines and reduced concentrations of Th2-type cytokines (IL-4 and IL-5), IL-10, and IL-17. The results of our study showed that vitamin D_3_ supplementation in patients with T1DM and T2DM reduced the concentrations of the inflammatory Th1-type cytokines and increased the levels of Th2-type cytokines.

**Conclusion:** The presence of two endocrine diseases, aggravated by decreased circulating levels of 25(OH)D, leads to disorders wherein the immune status is markedly changed. These decreased levels of 25(OH)D contribute to an autoimmune inflammatory process and to the progression of complications in addition to the metabolic disorders. A vitamin D intervention resulted in significant changes in the blood levels of 25(OH)D that are related to parameters of autoimmunity and glucose metabolism. Vitamin D_3_ supplementation should be considered for the prevention and treatment of combined endocrine pathology.

## Introduction

The incidence of a combination of endocrine diseases is highly prevalent worldwide. Diabetes mellitus (DM) with coinciding thyroid diseases is a common combination, affecting 17–30% of the cases in the clinical endocrinology practice in Ukraine (1, 2).

Type 1 diabetes (T1DM) is often associated with autoimmune diseases, such as autoimmune thyroid disease (ATD), celiac disease, autoimmune gastritis, pernicious anemia, and vitiligo (3). ATD is the most prevalent endocrinopathy among patients with diabetes (1, 4). The prevalence of ATD during the course of T1DM was as high as 60% of patients, 40% of whom additionally suffered from thyroid disorders including overt hypothyroidism (24%), subclinical hypothyroidism (8%), and hyperthyroidism (8%) (5). According to Krzewska et al. the ATDs Hashimoto's thyroiditis and Graves' disease, are the most prevalent autoimmune diseases in children and adolescents with T1DM. Their incidence is an estimated as 2–4-fold higher than those in the general population, with Hashimoto's thyroiditis the most common clinical form (14–28%) (4). T1DM is frequently associated with autoimmune endocrine and non-endocrine diseases and patients with T1DM are at a higher risk of developing several glandular autoimmune diseases. Familial clustering has been observed, suggesting a genetic predisposition. Various hypotheses of viral- and/or bacterial-induced pancreatic autoimmunity have been proposed; however, a definitive description of the autoimmune pathomechanism is still lacking (5, 6).

Furthermore, there is a deep underlying relationship between diabetes mellitus type 2 (T2DM) and thyroid dysfunction (6). Numerous studies have reported the complex intertwining of biochemical, genetic, and hormonal malfunctions characteristic of this pathophysiological association (4–7). The prevalence of thyroid disorder in a population affected with diabetes was 13.4% and appeared more prevalent (31.4%) in female T2DM patients compared to that in male T2DM patients (6.9%) (1).

Over the past 20 years a steady increase in the incidence of combined endocrine diseases has been observed in Ukraine (8). In addition to this disadvantageous epidemic trend is the substantial healthcare burden for Ukraine. The increasing prevalence of combined endocrine diseases most likely reflects the late health consequences of the Chernobyl disaster (April 26th, 1986), especially radioactive iodine that affected the thyroid gland. In the first decade after the Chernobyl disaster, the combination of diabetes with diffuse toxic goiter predominated; later, the prevalence of diabetes coinciding with thyroiditis increased significantly. The formation of nodules in the thyroid gland in older adults began to increase (9–11). According to Institute of Endocrinology and Metabolism data during the 5 years preceding the Chernobyl nuclear accident, a total of 59 cases of thyroid carcinoma were identified in the birth to 18 years age group (25 cases in those aged 14 years of less and 34 cases in adolescents aged 15–18 years). Between 1986 and 1997, there was a total of 577 of thyroid carcinomas in Ukrainian children and adolescents (358 and 219 cases, respectively). The largest number of cases occurred in patients living in areas of thyroid radiation doses of at least 0.50 Gr. Thyroid cancers developed after a short latent period, were more aggressive at presentation, and expressed regional (57.3%) or distant (14.5%) metastasis. Solid papillary cancers were present in 93.1% and coexisting chronic thyroiditis in 10.2% of cases (10).

Foley et al. focused on the long-term effects of the Chernobyl catastrophe, particularly as an unprecedented event that affected the endocrine system in individuals of all ages and which has provided data on the effects of radiation on humans, including the acceleration of thyroid cancer (12). The first years following the Chernobyl disaster were notable for several striking epidemiologic observations. First, a significant increase of incidence of pediatric thyroid cancer was reported in the two countries that were most contaminated by the release of radioiodine from the damaged nuclear reactor: Ukraine, the site of the Chernobyl plant, and Belarus, located directly to the north. The increased incidence of the thyroid cancer was particularly evidenced in individuals aged 0–4 years at the time of exposure and adults at the time of Chernobyl accident were markedly less affected. From the Ukrainian clinical perspective, the majority of thyroid carcinomas (more than 90%), noted in patients in their growth and maturation periods at the time of the Chernobyl accident, were diagnosed with papillary carcinoma (13).

The autoimmune pathology in clinical practice seems a difficult task for endocrinologists, with many unknowns. Modern laboratory diagnosis with high sensitivity allows the detection of autoantibodies in DM and autoimmune thyroiditis (AIT) and the monitoring of a wide spectrum of markers of the immune status of patients for diagnostic research and treatment. Unfortunately, during the treatment course, it is often very difficult or impossible to effectively reduce auto-aggression (1, 14).

T1DM and T2DM, as well as AIT in combination, have a constant tendency to progress the so-called “Chernobyl footprint” regions. As a result of both therapeutic difficulties and the relatively high prevalence of cases with these combined endocrine diseases in Ukraine, it is important to identify alternative methods to improve the treatment course and clinical outcomes. Among the promising agents and possible methods, proper vitamin D supply offers promise due to its low cost and easy accessibility.

25-hydroxyvitamin D [25(OH)D] is involved in the regulation of many physiological processes in the body and beneficial effects related to treatment with cholecalciferol were noted in immunodeficiency, cardiovascular disorders, anemia, diabetes, various pathologies of the liver, and gastrointestinal tract disorders, as well as for tuberculosis and malignant tumors of the breast and intestine (15, 16). Both genetic predisposition and environmental factors may contribute to the development of autoimmune diseases. Increased levels of immune inflammation markers may affect the course of immuno-endocrine pathology. A gradual development of the autoimmune inflammatory process, when combined with hypothyroidism and DM, may significantly contribute to the development of endothelial dysfunction and the consequent development of vascular complications. Therefore, the aim of our study was to determine the background concentrations of cytokines in patients with combined pathology–T1DM or T2DM and AIT in the context of vitamin D status. An additional objective of the study was to investigate the protective effect of vitamin D supplementation on the progression of the immune process.

## Patients and methods

### Study group

The study group consisted of 60 patients (21 males) aged ≥ 20 years with diabetes and AIT. All patients with AIT included in this study were euthyroid and on levothyroxine hormonal replacement therapy with thyroid-stimulating hormone (TSH) levels within the reference ranges. Among the 60 patients with AIT, 30 each had T1DM and T2DM. The TSH levels and levothyroxine dose did not change significantly during the study period. There were no statistically significant differences in the patients' body mass index (BMI) during the study period.

A separate group of 40 patients with T1DM or T2DM only comprised the control group. Patients who reported taking calcium and vitamin D supplements during the 6 months before the study were excluded. The study group as a whole was evaluated and treated at two in-patient departments of the Kyiv city Center of Endocrinology and Metabolism in Ukraine.

Table 1 describes the general characteristics of the patients included in the study and control groups.

**Table 1:** Characteristics of the patient and control group (mean ±*SD*).

	**T1DM** + **AIT**		**T2DM** + **AIT**		**T1DM**		**T2DM**	
	**Male**	**Female**	**Male**	**Female**	**Male**	**Female**	**Male**	**Female**
n	12	18	9	21	9	11	10	10
Age, yrs	36 ± 5	45 ± 7	57 ± 8	61 ± 9	33 ± 7	44 ± 6	57 ± 7	54 ± 7
BMI, kg/m^2^	30 ± 4	33 ± 4	36 ± 4	39 ± 4	30 ± 4	32 ± 5	37 ± 4	37 ± 6
AIT duration, yrs	6 ± 2	6 ± 2	4 ± 2	8 ± 2				
DM duration, yrs	9 ± 5	12 ± 5	9 ± 5	16 ± 4	10 ± 4	12 ± 5	10 ± 2	14 ± 3

*T1DM, diabetes mellitus type 1; T2DM, diabetes mellitus type 2; AIT, autoimmune thyroiditis; BMI, body mass index; DM, diabetes mellitus*.

## Methods

### Clinical assessment and laboratory investigations

The size and structure of the thyroid gland were evaluated by palpation and by ultrasound investigation. The evaluation of thyroid size by the palpation method was carried out in accordance with the WHO classification (2001) that includes three stages. According to the results of the ultrasound examination, goiter was diagnosed if the thyroid volumes in women and men exceeded 18 and 25 mL, respectively. Ultrasonography of the thyroid gland and volume determinations were performed using an HD 11 XE ultrasound apparatus (No. 453561262961, Philips, Amsterdam, the Netherlands) with an 8-MHz linear sensor. The volume of the thyroid lobes was calculated according to the formula proposed by Brunn (7):

$$\begin{array}{l} \text{V}=\left(\text{length}\times \text{width}\times \text{thickness}\right)\times 0.479 \end{array}$$

The functional status of the thyroid gland was evaluated based on the levels of thyroid-stimulating hormone (TSH), free thyroxine (fT4), and free triiodothyronine (fT3). The presence of AIT was confirmed by an increased thyroperoxidase (ATPO) titer.

The assay for 25(OH)D measured the total 25(OH)D, including both 25-hydroxyvitamin D_2_ and D_3_. The 25(OH)D concentration was assessed using a quantitative enzyme immunoassay kit (25-OH D IDS OCTEIA, Immunodiagnostik, Bensheim and Biomedica, Wien, Austria) with an intra-assay precision of <8% and inter-assay precision of <10%. The vitamin D status was assessed using the following criteria: 25(OH)D concentrations <75 nmol/L indicated insufficiency, while concentrations <50 nmol/L indicated vitamin D deficiency.

The glycated hemoglobin (HbA1c) level was determined by immuno-turbidimetry using standard test systems on an Advia 1800 biochemical analyzer, Siemens (Munich, Germany).

Determination of the concentrations of TSH, fT4, fT3 and antibodies to ATPO were performed by immunoassay analysis with standards on an Advia 1800 biochemical analyzer (Siemens, Munich, Germany).

The lipid spectrum of the blood was assessed by colorimetric (for cholesterol) and spectrophotometric (for triglycerides) methods on the Advia 1800 analyzer (Siemens, Munich, Germany).

The total calcium level was determined by colorimetric method on the Advia 1800 and ionized calcium by ion-selective method using the ion-selective electrolyte analyzer on an EasyLyte instrument (Medica Corporation, Bedford, MA, USA).

Determination of insulin concentration and parathyroid hormone (PTH) level was performed by immunoassay using standard sets of test systems on a Centaur biochemical analyzer (Siemens, Munich, Germany).

The levels of Th1-type cytokines (interferon [IFN]-γ, tumor necrosis factor [TNF]-α, interleukin [IL]-2, IL-6, IL-12), Th2-type cytokines (IL-4, IL-5), and IL-10 and IL-17 were measured by flow cytometry.

### Study protocol

Patients underwent traditional treatments for DM and hypothyroidism and received vitamin D_3_ supplements at a dose of 2,000–4,000 IU/day, depending on the blood levels of 25(OH)D and the presence or absence of chronic complications of diabetes (in particular, diabetic retinopathy) and obesity (16).

All patients with BMI <29.9 kg/m^2^ (24 kg/m^2^ for T1DM+AIT patients and 22 kg/m^2^ for T2DM+AIT patients) with 25(OH)D deficiency or insufficiency, as well as 14 patients with different grades of obesity, started vitamin D_3_ supplementation at a dose 4,000 IU/day. In cases that reached a 25(OH)D concentration of 72.5 nmol/L after the first course of supplementation, the dose was changed to 2,000 IU/day (about 10% of patients). This study used a certified vitamin D preparation with the highest bioavailability, since this preparation is a cholecalciferol protein complex. It was synthesized at the Institute of Biochemistry of Ukrainian National Academy of Medical Sciences.

The vitamin D_3_ preparations were prescribed for a period of 2 months (except for the summer period) at intervals of at least 3 months, with the course repeated twice each year.

Patients were examined after 10+/− 1 months with a re-examination of the levels of the metabolic and immune markers. Traditional hypoglycemic therapy in DM patients was not changed significantly during this trial as well as replacement levothyroxine therapy.

The results were analyzed using comparative analysis and variation statistics with calculation of the frequency characteristics of the parameters (P), the mean values, and the variability (standard deviation [SD]). The statistical significance of the differences the between treatment groups for the studied parameters was assessed using Wilcoxon-Mann-Whitney tests. *P* < 0.05 were considered statistically significant.

This study was carried out in accordance with the recommendations of the Ethics Committee of Bogomoletz National Medical University with written informed consent from all subjects. All subjects gave written informed consent in accordance with the Declaration of Helsinki. The protocol was approved by the Ethics Committee of Bogomoletz National Medical University.

## Results

### Baseline characteristics

The levels of metabolic markers in patients with combined endocrine disorders are shown in Table 2.

**Table 2:** Baseline values of metabolic markers and 25(OH)D concentration evaluated in patients with Diabetes Mellitus type 1, 2 and combined with Autoimmune Thyroiditis, (mean ± *SD*).

**Indicators**	**T1DM**	**T1DM + AIT**	**T2DM**	**T2DM + AIT**
Ca++ (mmol/L)	1.1 ± 0.3	1 ± 0.3	1.2 ± 0.3	1.1 ± 0.2
Ca total (mmol/L)	2.1 ± 0.3	2 ± 0.2	2.1 ± 0.4	1.9 ± 0.3
PTH (pmol/L)	6.4 ± 1.5	7.6 ± 1.6^*^	9.4 ± 1.8	13.5 ± 1.9^*^
25(OH) D (nmol/L)	49 ± 10	36 ± 9^*^	47 ± 7	34 ± 6.5^*^
Insulin (IU/mL)	9.2 ± 2.1	10.4 ± 2.7	16.6 ± 2.7	19.3 ± 2.5^*^
HOMA-IR	3,6 ± 0.5	4.3 ± 0.8^*^	6.8 ± 0.9	8.2 ± 1.4^*^
Triglycerides (mmol/L)	1.7 ± 0.6	2.5 ± 0.5^*^	2.0 ± 0.5	2.7 ± 0.7^*^
Fasting glucose (mmol/L)	8.8 ± 0.9	9.3 ± 1.1^*^	9.2 ± 1.3	9.5 ± 2.4
HbA_1_c, %	9.1 ± 1.7	9.7 ± 1.4	9.9 ± 1.4	10.3 ± 1.5
Cholesterol (mmol/L)	5.7 ± 1.3	6.5 ± 1.4^*^	6.4 ± 1.5	7.2 ± 1.3^*^

* *Significant difference between the T1DM/T2DM and T1DM+AIT/T2DM+AIT groups (p < 0.05)*.

### Follow-up characteristics

After vitamin D_3_ supplementation, the patient‘s metabolic and immune profile changed as shown in Table 3.

**Table 3:** Metabolic markers and 25(OH) D levels dynamic in patients with T1DM + AIT, T2DM + AIT after vitamin D_3_ supplementation, (M ± σ).

	**T1DM** + **AIT**		**T2DM** + **AIT**	
	**Before** **treatment**	**After** **treatment**	**Before** **treatment**	**After** **treatment**
Ca++ (mmol/L)	1.0 ± 0.3	1.3 ± 0.25	1.1 ± 0.2	1.28 ± 0.25
Ca total (mmol/L)	2.0 ± 0.2	2.4 ± 0.3^*^	1.9 ± 0.3	2.5 ± 0.4^*^
PTH (pmol/l)	7.6 ± 1.6	3.9 ± 1.71^*^	13.5 ± 1.9	7 ± 1.9^*^
TSH (mIU/l)	2.5 ± 0.2	2.4 ± 0.3	2 ± 0.2	2.2 ± 0.2
25(OH) D (nmol/L)	36 ± 9	72 ± 8^*^	34 ± 6.5	69 ± 7^*^
Insulin (IU/mL)	10.0 ± 3.0	5.3 ± 2.7^*^	19.0 ± 3.0	6 ± 0.6^*^
HOMA-IR	4.3 ± 0.8	2 ± 0.2^*^	8.2 ± 1.4	2 ± 0.6^*^
Triglycerides (mmol/L)	2.5 ± 0.5	1.4 ± 0.2^*^	2.7 ± 0.7	1.5 ± 0.8^*^
Fasting glucose (mmol/L)	9.3 ± 1.1	8.3 ± 1.3	9.5 ± 2.4	8 ± 0.6^*^
HbA_1_c, %	9.7 ± 1.4	8.5 ± 0.9^*^	10.3 ± 1.5	8.6 ± 1^*^
Cholesterol (mmol/L)	6.5 ± 1.4	4.9 ± 0.5^*^	7.2 ± 1.3	6.1 ± 1.3

* *Significant difference from the indicator before treatment (p < 0.05)*.

### Immune status before and after treatment

We evaluated the effects of vitamin D_3_ supplementation in patients with both DM and AIT according to the level of Th1-type cytokines (INF-γ, TNF-α, IL-2, IL-6, IL-12) and Th2-type cytokines (IL-4, IL-5, IL-10, IL-17).

Our analyses revealed immunologic shifts in patients with combined endocrine pathology as well as their changes after vitamin D_3_ supplementation (Table 4).

**Table 4:** Comparative evaluation of cytokines, in patients with combined endocrine pathology (DM + AIT) and DM patients before and after vitamin D treatment (mean ± *SD*).

**Cytokines, pg/mL**	**T1DM** + **AIT**		**T2DM** + **AIT**	
	**Before** **treatment**	**After** **treatment**	**Before** **treatment**	**After** **treatment**
IFN-ɤ	220 ± 50	55 ± 10^*^	270 ± 60	58 ± 11^*^
TNF-α	125 ± 16	56 ± 9^*^	183 ± 25	58 ± 9^*^
IL-2	113 ± 16	29 ± 7^*^	124 ± 14	22 ± 8^*^
IL-6	150 ± 35	30 ± 11^*^	180 ± 25	52 ± 12^*^
IL-12	180 ± 50	56 ± 17^*^	130 ± 20	27 ± 11^*^
IL-4	16 ± 5	160 ± 22^*^	25 ± 8	170 ± 30^*^
IL-5	56 ± 12	140 ± 28^*^	20 ± 5	120 ± 24^*^
IL-10	28 ± 16	98 ± 40^*^	10 ± 3	72 ± 21^*^
IL-17	45 ± 15	138 ± 33^*^	40 ± 12	118 ± 22^*^

* *Significant difference with the indicator before treatment (p = 0.0001, Wilcoxon-Mann-Whitney tests)*.

The concentrations of INF-γ in patients with T1DM and AIT and decreased T2DM and AIT by 3.9-fold and 5.2-fold compared to the concentrations before treatment. The levels of TNF-α after treatment with vitamin D_3_ were significantly reduced by 2.3-fold in patients with a combination of T1DM and AIT, and by 3.2-fold in patients with T2DM and AIT. Similar changes were observed for IL-2, with a 3.9-fold decrease in blood concentration after vitamin D treatment in patients with T1DM and AIT and a 5.7-fold decrease with T2DM and AIT. The blood concentration of IL-6 decreased by 4.9-fold and 3.5-fold due to vitamin D_3_ supplementation in patients with T1DM and AIT and T2DM and AIT, respectively. In patients with T1DM and AIT, the level of IL-12 after vitamin D_3_ supplementation decreased significantly by 2.4-fold and by 4.8-fold in patients with T2 DM and AIT.

In patients with T1DM and AIT, the level of IL-4 in the blood was increased significantly by 10-fold and by 6.7-fold in patients with T2DM and AIT following vitamin D_3_ supplementation. As a result of vitamin D_3_ supplementation, the level of IL-5 increased by 2.5-fold and 5.9-fold in patients with T1DM and AIT and T2DM and AIT, respectively. In patients with T1DM and AIT, the level of IL-10 increased by 3.5-fold and by 6.6-fold in patients with T2DM and AIT. After vitamin D_3_ supplementation, the concentration of IL-17 increased significantly by 3-fold in patients with T1DM and AIT and 2.9-fold in patients with T2DM and AIT.

To detect the presence or absence of relationships between change in HbA1c level and immunological parameters, we conducted a comparative assessment of the dynamics of cytokines on the background of treatment with vitamin D in groups with a small change in the level of HbA1c (under median) compared to that in the group of patients with significant HbA1c improvement dynamics (above median). This analysis was performed separately for patients with T1DM+AIT and T2DM+AIT (Tables 5, 6).

**Table 5:** Comparison of the cytokine dynamics in groups with different levels of HbA1c reduction in patients with T1DM+AIT.

**Cytokines, pg/mL**	**HbA1c**<**1.2%**		**Δ** (95%CI)^*^	**HbA1c**> **1.2%**		**c** **(95%CI)**	P(Δ)^**^
	**Before** **treatment**	**After** **treatment**		**Before** **treatment**	**After** **treatment**
IFN-ɤ	220 ± 60	58 ± 7.3	-164.1 (127–201.3)	208 ± 41	51 ± 12	-156.4 (123.7–189)	0.76
TNF-α	124 ± 13	25 ± 9.0	-98.5 (89–108)	127 ± 21	26 ± 9.0	-101.4 (84.2–118.5)	0.94
IL-2	113 ± 13.0	29 ± 8	-83.4 (74.2–92.6)	114 ± 20	27 ± 6	-86.1 (70.4–101.9)	0.25
IL-6	150 ± 30	33 ± 12	-115.1 (95.6–134.5)	150 ± 42	26 ± 7	-124.8 (92.3–157.4)	0.99
IL-12	190 ± 60	61 ± 16	-126.4 (88.4–164.4)	170 ± 37	48 ± 14	-123.7 (93.7–153.6)	0.82
IL-4	17.6 ± 5.3	160 ± 26	142.4 (126.6–158.3)	14 ± 3.3	165 ± 15.3	150.6 (138.8–162.5)	0.11
IL-5	55 ± 13	136 ± 34	81.7 (60–203.5)	58 ± 12	150 ± 15	92.5 (77.9–107)	0.12
IL-10	25 ± 16	100 ± 46	76.1 (46.7–105.3)	33 ± 17	93 ± 30	60.1 (33.9–86.2)	0.7
IL-17	44 ± 15	145 ± 35	100.2 (77.5–122.8)	48 ± 15	130 ± 30	80.2 (54.2–106.2)	0.13

* Δ (95%CI): absolute dynamics of indicators before/after treatment, 95% confidence interval.

** *P (Δ): evaluation of the statistical significance of the difference between the indicator dynamics in patients with minimal and significant ΔHbA1c changes (<1.2 and >1.2%, respectively) (Wilcoxon-Mann-Whitney test)*.

**Table 6:** Comparison of the cytokine dynamics in groups with different levels of HbA1c reduction in patients with T2DM+AIT.

**Cytokines, pg/mL**	**HbA1c**<**1.7%**		**Δ** (95%CI)^*^	**HbA1c** > **1.7%**		**c** **(95%CI)**	P (Δ)^**^
	**Before** **treatment**	**After** **treatment**		**Before** **treatment**	**After** **treatment**
IFN-ɤ	290 ± 65	62 ± 10.5	-226.9 (187.5–266.2)	240 ± 52	51 ± 9.6	-189.0 (148.6–229.3)	0.76
TNF-α	175 ± 27	33 ± 7.6	-141.7 (124.8–158.6)	195 ± 13.7	36 ± 12	-159.3 (145.3–173.3)	0.11
IL-2	130 ± 12	25 ± 7.3	-105.6 (97.5–113.7)	115 ± 13	17 ± 7.6	-98.3 (87.0–109.4)	0.26
IL-6	177 ± 30	54 ± 14	-123.6 (103.8–143.4)	180 ± 15	48 ± 7.4	-132.9 (119.9–145.9)	0.62
IL-12	130 ± 17	29 ± 11.3	-100.8 (88.6–112.9)	130 ± 14.8	26 ± 12	-104 (89.5–118.4)	0.23
IL-4	22.5 ± 7.1	180 ± 21	156.7 (143.3–169.9)	28.6 ± 7.7	150 ± 35	120.8 (93.8–147.6)	0.09
IL-5	20 ± 6	106 ± 16	85.6 (75.5–95.6)	20 ± 4.7	140 ± 20.8	119 (102.8–135.2)	0.44
IL-10	10.8 ± 3.2	70.5 ± 25	59.7 (44.7–74.6)	11 ± 3.3	74 ± 13	63.2 (73.2–53.1)	0.76
IL-17	120 ± 27	77 ± 7.7	-42.8 (26.0–59.6)	116 ± 12.6	81 ± 13	-34.9 (21.1–48.6)	0.25

* Δ (95%CI): absolute dynamics of the indicators before/after treatment, 95% confidence interval.

** *P (Δ): evaluation of the statistical significance of the difference between the indicator dynamics in patients with minimal and significant ΔHbA1c changes (<1.7 and >1.7%, respectively) (Wilcoxon-Mann-Whitney test)*.

The minimum decrease of HbA1c in the T1DM+AIT group was 0.49% (maximum: −1.9%, median: 1.2%). The patients in this group were divided into two subgroups: HbA1c dynamics <1.2 and >1.2%. The main hypothesis was based on comparing the cytokine dynamics in groups with different levels of HbA1c reduction indicating moderate and significant improvements. There was no statistically significant advantage in the dynamics of cytokines in the group with higher dynamics (ΔHbA1c >1.2%) compared to that in the group with minor changes (ΔHbA1c <1.2%) (*p* > 0.05).

The minimum HbA1c decrease in T2DM+AIT patients was 0.93% (maximum: −2.46%, median: −1.7%). The patients in this group were divided into two subgroups: HbA1c dynamics <1.7 and >1.7%. The main hypothesis was based on comparing the cytokine dynamics in groups with different levels of HbA1c changes, indicating moderate and significant improvement. There was no statistically significant difference in the cytokine dynamics in the group with higher dynamics (ΔHbA1c >1.7%) compared to that in the group with minor changes (ΔHbA1c <1.7%) (*p* > 0.05).

There was a trend toward greater cytokine dynamics in groups with higher dynamics (“c”), but the absence of a statistically significant difference in the cytokine dynamics in groups with minor changes in Δ HbA1c compared to a group of patients with a significant improvement in HbA1c levels does not indicate a priori determinism of cytokine changes due to changes in HbA1c.

Thus, the results of vitamin D_3_ supplementation in patients with a combination of DM and AIT showed that vitamin D_3_ has a positive effect on the balance of cytokines in the blood of patients with a combined endocrine pathology.

## Discussion

25(OH)D deficiency in patients with DM is accompanied by higher levels of glycemia and glycated hemoglobin. 25(OH)D deficiency also causes the early development of complications of DM. As highlighted elsewhere, there is a significant inverse association between serum 25(OH) D and HbA1c. The percentage of 25(OH)D deficiency/insufficiency in the population and the growth of DM prevalence suggest that vitamin D_3_ supplementation can improve overall health. There is so much benefit from supplementation with vitamin D or by adding natural vitamin D rich food in the diet, including physical activities with possible sun light exposure. Advising patients with higher HbA1c to get tested for 25(OH)D levels and correct any deficiency/insufficiency if found may result in better blood glucose control and benefit the patient's overall health (7, 17–19). The important result we find from this study is a significant reduction in HbA1c as 25(OH)D levels increased.

DM with coinciding AIT is a serious clinical problem in Ukraine. The combination of these two diseases may affect the severity of the clinical course and therapeutic difficulties may be exacerbated by 25(OH)D deficiency, another well-documented public health challenge in Ukraine (8, 20).

We observed the most severe and resistant decompensation of carbohydrate metabolism on a background of 25(OH) D deficit in patients with T2DM combined with AIT. The homeostatic model assessment of insulin resistance (HOMA-IR) index in patients with T1DM or T2DM combined with AIT was higher than normal, especially in patients with T2DM with AIT. The indicators of fat metabolism, such as triglyceride and cholesterol levels, were higher than normal in patients with combined endocrine disorders compared to those in DM patients. Similar results were noted in other papers (21, 22).

The results of our study revealed a reduction in the total and ionized calcium levels before vitamin D supplementation (at baseline) in all patients, regardless of the type of diabetes. In addition, a compensatory increased PTH level was observed in patients with DM + AIT with coinciding reduced 25(OH)D concentration (less than 75.0 nmol/L). Some authors have postulated the possibility of a relationship with coexisting 25(OH)D concentrations (3, 15, 21).

Vitamin D_3_ supplements were prescribed in accordance with blood levels of 25(OH)D at a dose of 2,000–4,000 IU/day according to the Endocrine Society clinical practice guidelines (16).

The experimental data on the hydroxylation of vitamin D in the liver, the involvement of reticulocytes in the deposition of 25(OH)D, and the results of vitamin D supplementation in the clinic demonstrating the possibility of maintaining the required concentration of 25 (OH) D in the blood for 2–3 months; even after discontinuing vitamin D supplementation, we found it possible to use vitamin D in the exchange rate regimen. We also observed a satisfactory compliance among patients administered vitamin D (29, 30).

The results of vitamin D_3_ supplementation in the complex therapy of patients with DM indicated that the normalization of serum calcium level is associated with a decrease in PTH level. Moreover, an increase in 25(OH)D concentration resulted in improvements in carbohydrate metabolism, as assessed by fasting glucose levels in blood and HbA1c level.

25(OH)D deficiency has been associated with autoimmune disturbances, which and may be improved with vitamin D_3_ supplementation (23, 24). In patients with T1DM and T2DM and low 25(OH) D concentrations, the background concentration of Th1-profile cytokines was increased and that of the Th2-profile cytokines was reduced, leading to an imbalanced immune status that supported the autoimmune inflammatory process and created conditions for disease progression (25–28).

There is a lack of similar studies of patients with only DM or only AIT. Moreover, the results were contradictory. In some of studies no significant changes in HbA1c or insulin sensitivity in DM patients after Vitamin D repletion were found. (31, 32), in other studies there was an information that Vitamin D_3_ supplement improved HbA1C and glycemic parameters in T1DM and T2DM patients. (33, 34). These previous studies focused on the correction of disturbed metabolism in patients with types 1 and 2 DM by vitamin D_3_ supplementation.

There is little information on AIT in the literature. Patients with vitamin D deficiency/insufficiency have generally been studied in correlation with serum anti-TPO thyroid antibodies levels. In all studies, vitamin D supplementation led to a decrease in serum anti-TPO levels. “25(OH)D deficiency may be related to pathogenesis of AIT and that its supplementation could contribute to the treatment of patients with AIT,” “25(OH)D level is an independent factor affecting the presence of TPOAb in AIT. The causal effect of 25(OH)D deficiency to AIT is to be elucidated” (35, 36).

We found that the combination of AIT and DM significantly disturbed the immune status. The levels of the proinflammatory Th1-type cytokines were increased and the levels of Th2-profile cytokines were decreased. Our comparative analysis showed that the absence of a statistically significant difference in the dynamics of cytokines in groups with minor changes in ΔHbA1c compared to that in patients with a significant improvement in HbA1c levels indicates an absence of a priori determinism of cytokine changes due to changes in HbA1c levels.

The results of our study show that vitamin D_3_ supplementation in T1DM+AIT and T2DM+AIT patients reduced the concentration of inflammatory Th1 cytokines (INF-γ, TNF-α, IL-2, IL-6, and IL-12) and increased levels of anti-inflammatory Th2-profile cytokines (IL-4, IL-5) and IL-10 and IL-17. Given the association of proinflammatory cytokines with the development of chronic complications of DM, a decrease in proinflammatory cytokines and an increase in anti-inflammatory cytokines after vitamin D_3_ supplementation may retard the development of chronic diabetic complications.

## Conclusion

Patients with combined endocrine diseases (DM + AIT) accompanied with decreased vitamin D status had significantly increased Th1-type cytokine levels and significantly decreased Th2-type cytokine levels. This finding indicates the presence of immune status disorders in patients, which supports the autoimmune inflammatory process and creates conditions for disease progression. The results of the present study indicate that the combination of endocrine diseases with decreased circulating levels of 25(OH)D may amplify metabolic disorders and generally contribute to early complications in DM. Vitamin D_3_ supplementation in DM + AIT patients leads to the normalization of carbohydrate, mineral, and lipid metabolism to reduce the levels of pro-inflammatory cytokines, which may contribute to the effectiveness of the treatment of combined endocrine diseases.

Taking into consideration our results demonstrating the prevalence of 25(OH)D deficiency in these patients, vitamin D_3_ supplementation should be recommended in patients with combined endocrine pathology in order to correct metabolic and immunological disorders.

## Ethics statement

This study was carried out in accordance with the recommendations of the Ethics Committee of Bogomoletz National Medical University with written informed consent from all subjects. All subjects provided written informed consent in accordance with the Declaration of Helsinki. The protocol was approved by the Ethics Committee of Bogomoletz National Medical University.

## Author contributions

YK conceived the idea for the study, contributed to the design of the research, and collected the data. YK and MB analyzed the data and wrote the paper. All authors edited and approved the final version of the manuscript.

### Conflict of interest statement

The authors declare that the research was conducted in the absence of any commercial or financial relationships that could be construed as a potential conflict of interest. The handling Editor and reviewers WG & MH declared their involvement as co-editors in the Research Topic, and confirm the absence of any other collaboration.
